# Publisher Correction: Exocyst dynamics during vesicle tethering and fusion

**DOI:** 10.1038/s41467-019-08322-x

**Published:** 2019-01-15

**Authors:** Syed Mukhtar Ahmed, Hisayo Nishida-Fukuda, Yuchong Li, W. Hayes McDonald, Claudiu C. Gradinaru, Ian G. Macara

**Affiliations:** 1 Department of Cell and Developmental Biology, Vanderbilt University School of Medicine, Nashville, TN 37240 USA; 2 Department of Biochemistry and Molecular Genetics, Ehime University Graduate School of Medicine, Toon, Ehime, 7910295 Japan; 3 Department of Hepato-Biliary-Pancreatic and Breast Surgery, Ehime University Graduate School of Medicine, Toon, Ehime, 7910295 Japan; 4 Department of Physics, University of Toronto, Toronto, ON M5S 1A7 Canada; 5 Department of Chemical & Physical Sciences, University of Toronto Mississauga, Mississauga, ON L5L 1C6 Canada; 6 Department of Biochemistry, Vanderbilt University School of Medicine, Nashville, TN 37240 USA; 7 Present Address: Department of Genome Editing, Institute of Biomedical Sciences, Kansai Medical University, Hirakata, 5731010 Japan

Correction to: *Nature Communications* 10.1038/s41467-018-07467-5; published online 03 December 2018

The original version of this Article contained errors in the author affiliations.

Affiliation 2 incorrectly read ‘Department of Biochemistry and Molecular Genetics and Breast Surgery, Ehime University Graduate School of Medicine, 7910295, Japan’ and affiliation 3 incorrectly read ‘Department of Hepato-Biliary-Pancreatic and Breast Surgery, Ehime University Graduate School of Medicine, Matsuyama 7910295, Japan.’

These errors have now been corrected in both the PDF and HTML versions of the Article.

