# Supplementary material for: Exocyst dynamics during vesicle tethering and fusion
Source: Nat Commun. 2018 Dec 3;9:5140. doi: 10.1038/s41467-018-07467-5 (PMC6277416; doi:10.1038/s41467-018-07467-5)
Supplement: Supplementary file 1 — Supplementary Information [file 41467_2018_7467_MOESM1_ESM.pdf]

Supplementary information

Exocyst Dynamics During Vesicle Tethering and Fusion

Syed Mukhtar Ahmed[1*], Hisayo Nishida-Fukuda[1,2,3,§], Yuchong Li[4,5], W. Hayes McDonald[6], Claudiu Gradinaru[4,5] and Ian G. Macara[1*]

Supplementary documents include: 7 supporting and additional data figures, 1 figure showing original immunoblots, and 1 supporting table showing peptide counts for proteins identified by mass spectrometry data shown in Fig 1E, and a supporting table of reagents described in methods.

[1]Department of Cell and Developmental Biology, Vanderbilt University School of Medicine, Nashville, TN, 37240, USA, [2]Department of Biochemistry and Molecular Genetics and [3]Department of Hepato-Biliary-Pancreatic and [3]Breast Surgery, Ehime University Graduate School of Medicine, Japan, [4]Department of Physics, University of Toronto, Toronto, ON, M5S 1A7, Canada, [5]Department of Chemical & Physical Sciences, University of Toronto Mississauga, Mississauga, ON, L5L 1C6, Canada, [6]Department of Biochemistry, Vanderbilt University School of Medicine, Nashville, TN, 37240, USA.
[§]Current address: Department of Genome Editing, Institute of Biomedical Sciences, Kansai, Medical University, Japan
*Corresponding author

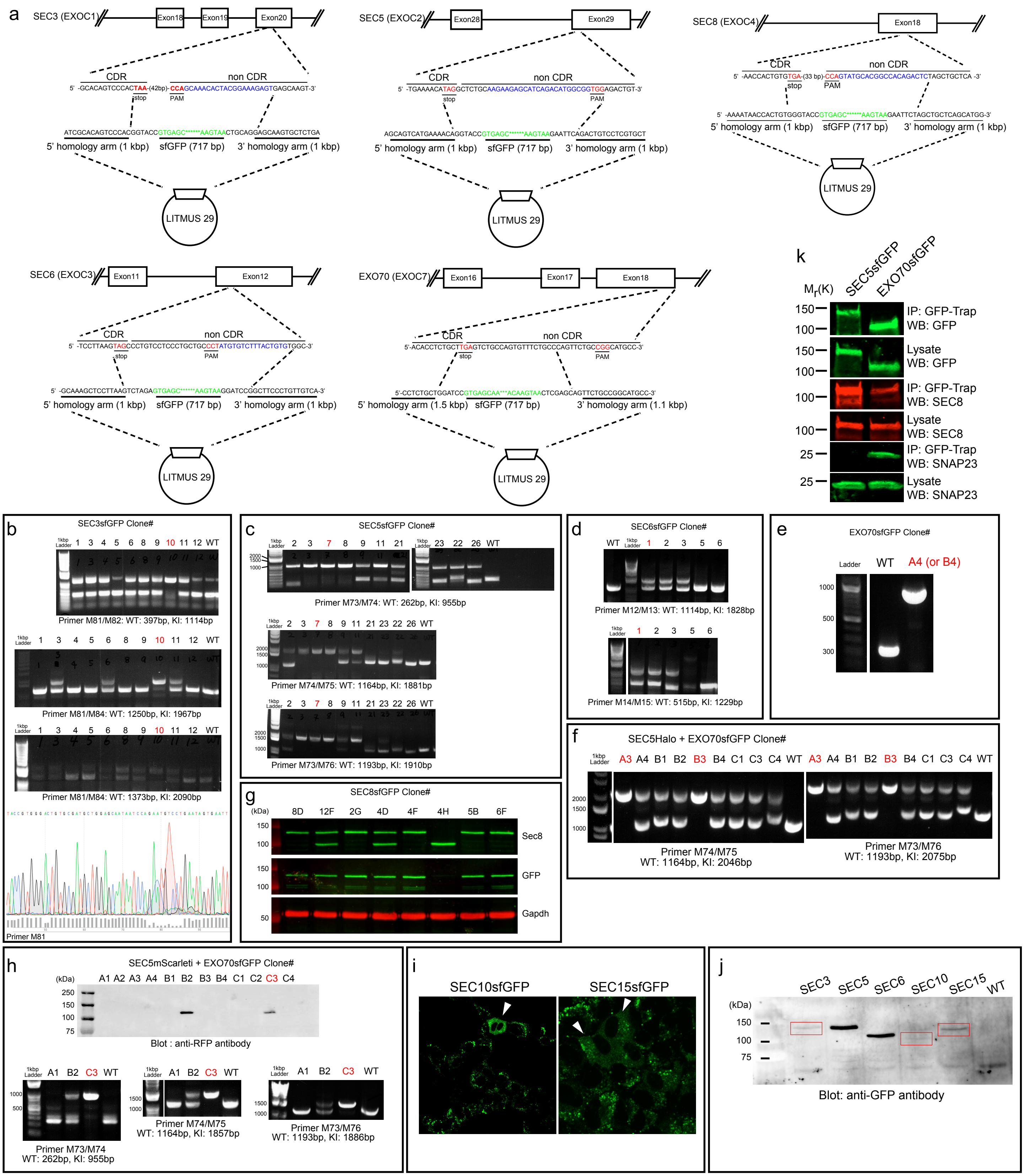

Supplementary Figure 1. Design and Validation of Gene-Edited Exocyst Cell Lines. (a) Schematic of designs for targeting vectors to insert sfGFP at the C-terminus of exocyst subunits SEC3, SEC5, SEC6 and EXO70 genes. Targeting vector deletes the STOP codon and PAM sequence. **(b)** Genotypes of SEC3-GFP clones using primer sets listed in Supplemental Table 2. Bottom panel shows sequencing of PCR product of the C-terminal region of clone 10. **(c-e)** Genotypes of SEC5-GFP, SEC6-GFP and EXO70-GFP clones as indicated. **(f)** Genotype of SEC5-Halo/EXO70-GFP double knock-in clones as indicated. **(g)** Western blot of SEC8-GFP clones using either anti-SEC8 or anti-GFP antibodies. GAPDH antibodies used to assess loading. **(h)** Western blot analysis and PCR genotyping of SEC5-mScarlet/EXO70-GFP double knock-in clones. SEC5-mScarlet expression was assessed using anti-RFP antibodies. **(i)** Confocal images of SEC10-GFP and SEC15-GFP after gene editing and FACS. White arrowheads indicated cells with successful incorporation of sfGFP. **(j)** Western blot analysis of SEC3-GFP, SEC5-GFP, SEC6-GFP, SEC10-GFP and SEC15-GFP cell lines using anti-GFP antibodies. WT = wild type parental cells. **(k)** SEC5-GFP or EXO70-GFP pulldown using GFP-Trap beads followed by immunoblot analysis to determine binding of native SEC8 (anti-SEC8 antibody) and SNAP23 (anti-SNAP23 antibodies). Pulldowns of SEC5-GFP or EXO70-GFP were assessed using anti-GFP antibodies.

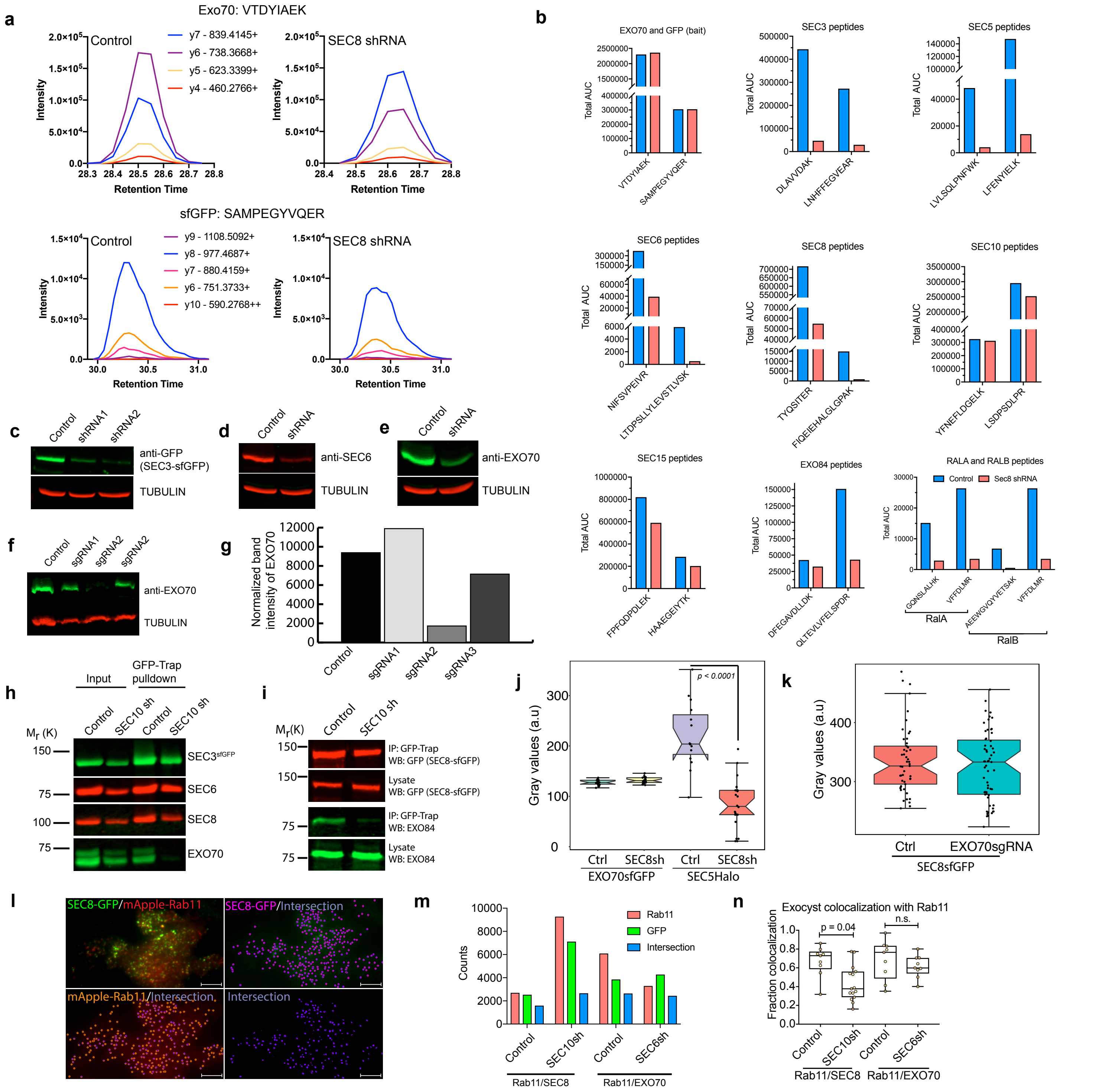

Supplementary Figure 2. Mammalian Exocyst Subunit Connectivity. (a) EXO70-GFP capture using GFP-Trap nanobodies. Shown are representative MRM spectra of intensity peaks features of the indicated EXO70 and sfGFP peptides in the control or SEC8 shRNA treated cells. **(b)** Quantifications of the area under the curve (AUC) for the indicated peptides for each protein species shown. Control pull-downs are denoted in blue whereas SEC8 depleted conditions are designated in red. **(c)** Knockdown efficiencies of two independent shRNAs targeting SEC3-GFP. α-Tubulin used as loading control. **(d)** Knockdown efficiency of SEC6 shRNA. **(e)** Knockdown efficiency of EXO70 shRNA. **(f)** Knockout efficiency with three independent guide RNAs targeting EXO70 gene loci in Exon1. **(g)** Quantification of panel F. **(h)** GFP-Trap pull-down of endogenous SEC3-GFP from untreated or SEC10 shRNA treated NMuMG cells. Western blots were immunoblotted with anti-GFP to detect SEC3-GFP, or with anti-SEC6, anti-SEC8 and anti-EXO70 antibodies to assess co-precipitations of unlabeled exocyst subunits. **(i)** GFP-Trap pull-down of endogenous SEC8-GFP from untreated or SEC10 shRNA treated NMuMG cells. Blots were probed with anti-GFP to detect SEC8-GFP, or with anti-EXO84 antibodies to assess amount of co-precipitation of endogenous unlabeled EXO84. **(j)** Quantification of fluorescence intensities from TIRFM images of SEC5-Halo and EXO70-GFP double knock-in cells treated with Control or SEC8 shRNAs. SEC5-Halo was labeled with JF585 Halo ligand. **(k)** Fluorescence intensity quantifications of SEC8-GFP from TIRFM images in untreated or EXO70 knockout cells. **(l)** Example of SEC8-GFP and mApple-Rab11 coincidence. Cells were transduced with mApple-Rab11 lentivirus. Spot diameters in the range of 0.30-0.35μm was used to identify GFP and Rab11 and the fraction of the particle that are both red and green were determined using NIS Elements spot detection algorithm. Scale bar = 5μm. **(m)** Total number of particles analyzed for Rab11, exocyst subunits and fraction that co-localize. **(n)** Quantification of the fraction of exocyst subunits SEC8-GFP or EXO70-GFP that coincide with mApple-Rab11. Ordinate axis = intersection/GFP. Cells were treated with scrambled, Sec10 or Sec6 targeting hairpins. Center lines show the medians; box limits indicate the 25th and 75th percentiles as determined by R software; whiskers extend 1.5 times the interquartile range from the 25th and 75th percentiles, data points are plotted as dots. Statistical significance was assessed using one-way ANOVA followed by Scheffe's multiple comparison tests.

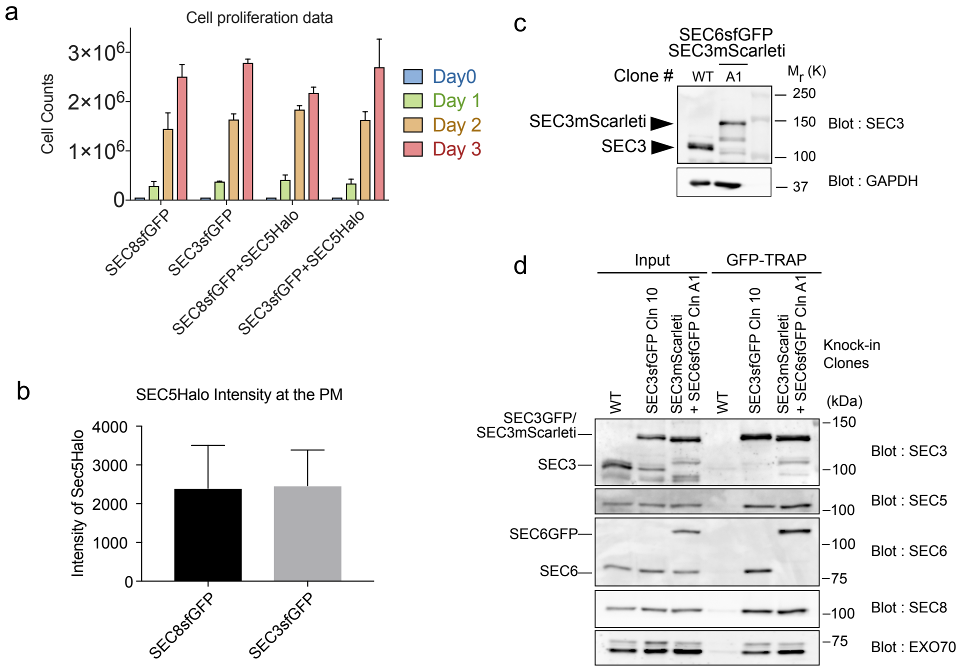

Supplementary Figure 3. Functionality of Exocyst Knock-in Cell-Lines. (a) Cell proliferation of SEC8-GFP, SEC3-GFP, SEC8-GFP + SEC5-Halo and SEC3-GFP + SEC5-Halo knock-in cells over three days. Error bars indicate s.d. Statistical analysis was using one-way ANOVA to compare groups for each day. **(b)** Comparison of SEC5-Halo fluorescence intensity at the bottom PM (PM) in double knock-in cells. Intensities were measured using TIRFM. Halo tag was labeled with JF585-HTL. **(c)** Western blot analysis of SEC3-Scarlet+ SEC6-GFP double knock-in cells. Clone A1 is homozygous for SEC3. WT = wild-type parental NMuMG cells. **(d)** GFP-Trap pull-down and western blots analysis from double knock-in cell lines expressing SEC3-GFP, or SEC3-Scarlet + SEC6-GFP. WT = wildtype cells, Cln = clone.

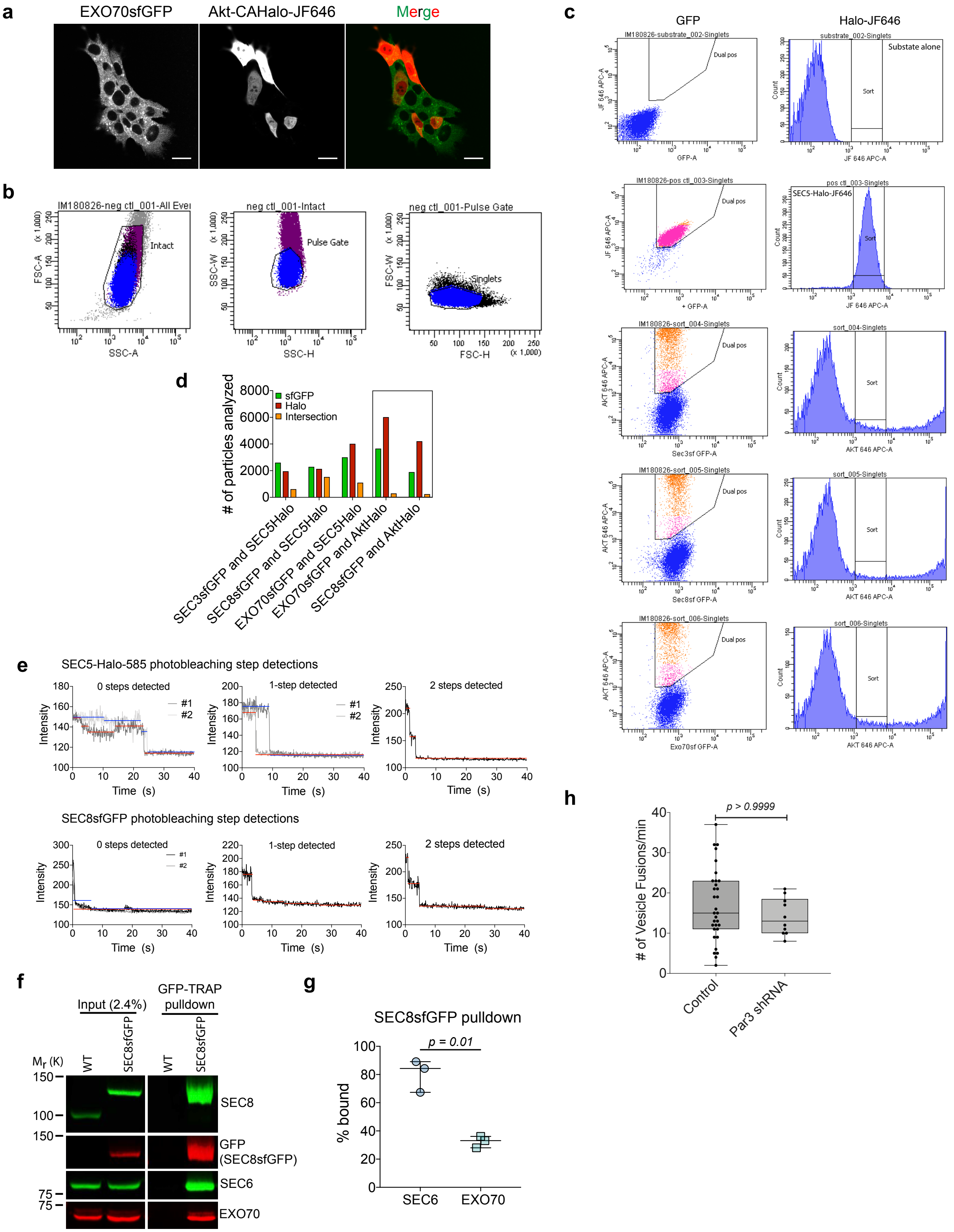

Supplementary Figure 4. Fractional Interactions of Cellular Pools of Exocyst subunits. (a) AKT-Halo was expressed in EXO70-GFP cells as a negative control for interactions. Halotag was labeled with JF-646 HTL and and imaged to look at the distribution of expression prior to cell sorting. Scale bar = 20µm. **(b)** Gating used for cell sorting in panel c. **(c)** SEC3-GFP, SEC8-GFP and EXO70-GFP knock-in cells were transduced with lentivirus expressing Akt-Halo, and sorted for similar expressed levels than SEC5-Halo. Halo was labeled with JF-646 HTL. **(d)** Graph shows number of molecules analyzed in the data shown in Figure 5d. **(e)** Representative images of 0, 1 or 2 photobleaching steps detected by the step detection algorithm. **(f)** Representative experiment where SEC8-GFP was captured using GFP nanobodies and immunoblotted with anti-SEC8, anti-GFP, anti-SEC6 or anti-EXO70 antibodies. Blots on the left show 2.4% input of the total lysate and the blot of the right shows pulldown with nanobodies. **(g)** Quantification of the percentage of SEC6 and EXO70 bound to SEC8 from 3 independent experiment experiments. **(h)** Number of Vamp2-pHluorin fusions at the bottom of cells in control or Par3 depleted NMuMG cells assessed using TIRF microscopy. *p* value calculated using Student's t-test.

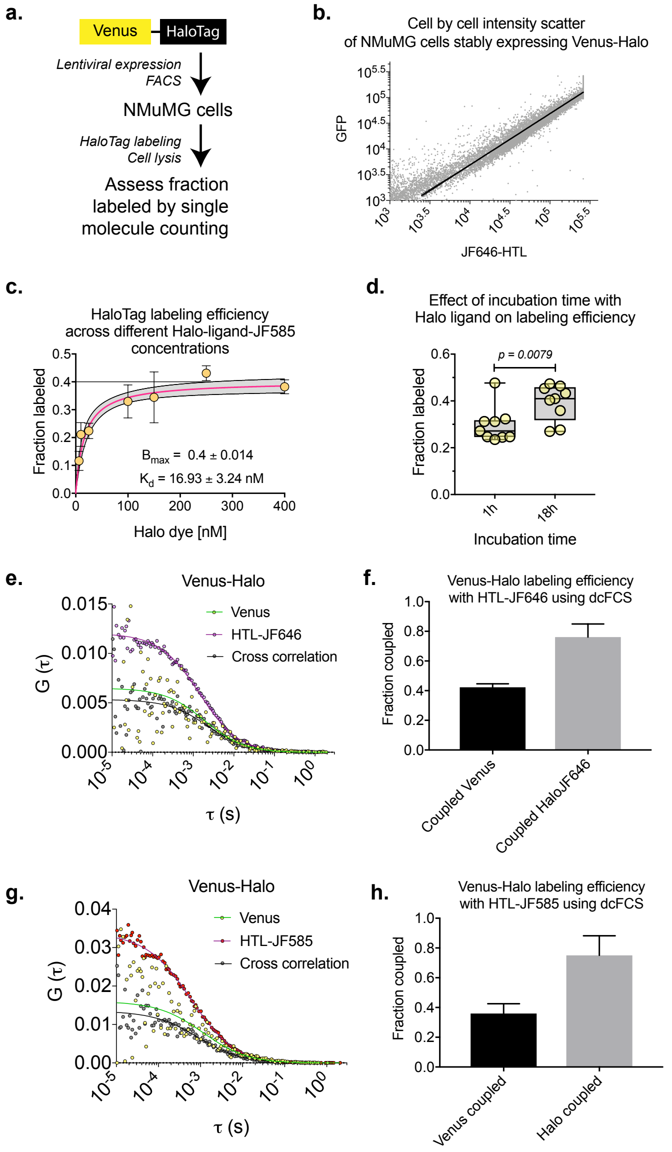

Supplementary Figure 5. HaloTag Labeling Efficiency. (a) Schematic showing experimental design to determine Halo labeling efficiency in NMuMG cells. Halo fused to the C-terminus of of YFP was expressed in NMuMG cells using lentiviral expression system, and subsequently sorted for positive cells. **(b)** Correlation between fluorescence intensities of YFP and Halo labeled with JF-646-HTL. **(c)** Halo labeling efficiency across different concentrations (6.25, 25, 100, 150, and 400 nM) of HaloTag ligand conjugated to JF585 dye in NMuMG cells for 1.5h. **(d)** Halo labeling efficiency after NMuMG cells were labeled with 100nM of HaloTag ligand conjugated to JF585 for 1h or 18h. **(e)** Dual-color FCCS measurement of Venus-Halo labeled with 150nM HTL-JF646 for 2h in NMuMG cells. **(f)** Quantification of the fraction of Venus-Halo that are labeled with HTL-JF646. **(g)** Dual-color FCCS measurement of Venus-Halo labeled with 150nM HTL-JF585 for 2h in NMuMG cells. **(h)** Quantification of the fraction of Venus-Halo that are labeled with HTL-JF585.

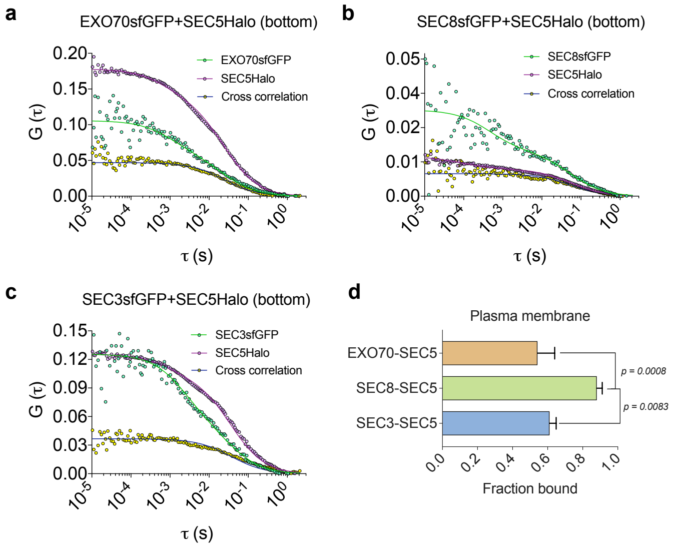

Supplementary Figure 6. Dual-Color FCCS Measurements of Exocyst Subunits near the base of the cells. (a) SEC5-Halo and EXO70-GFP, **(b)** SEC5-Halo and SEC8-GFP and **(c)** SEC5-Halo and SEC3-GFP FCS measurements near the base of the cells. **(d)** Statistics of fraction of GFP-tagged exocyst subunits near the bottom membrane of the cells. SEC8-GFP+SEC5-Halo: 64% ± 2.2%, SEC3-GFP+SEC5-Halo: 44%   2.6%, EXO70-GFP+SEC5-Halo: 39% ± 7.0% (mean ± s.e.m.). These numbers are probably underestimates as exocysts on the membrane are less diffusive. Also the measurements likely include both plasma membrane bound fraction as well as cytoplasmic fraction. HaloTag was labeled using JF646 Halo ligand (200 nM for 1.5h).

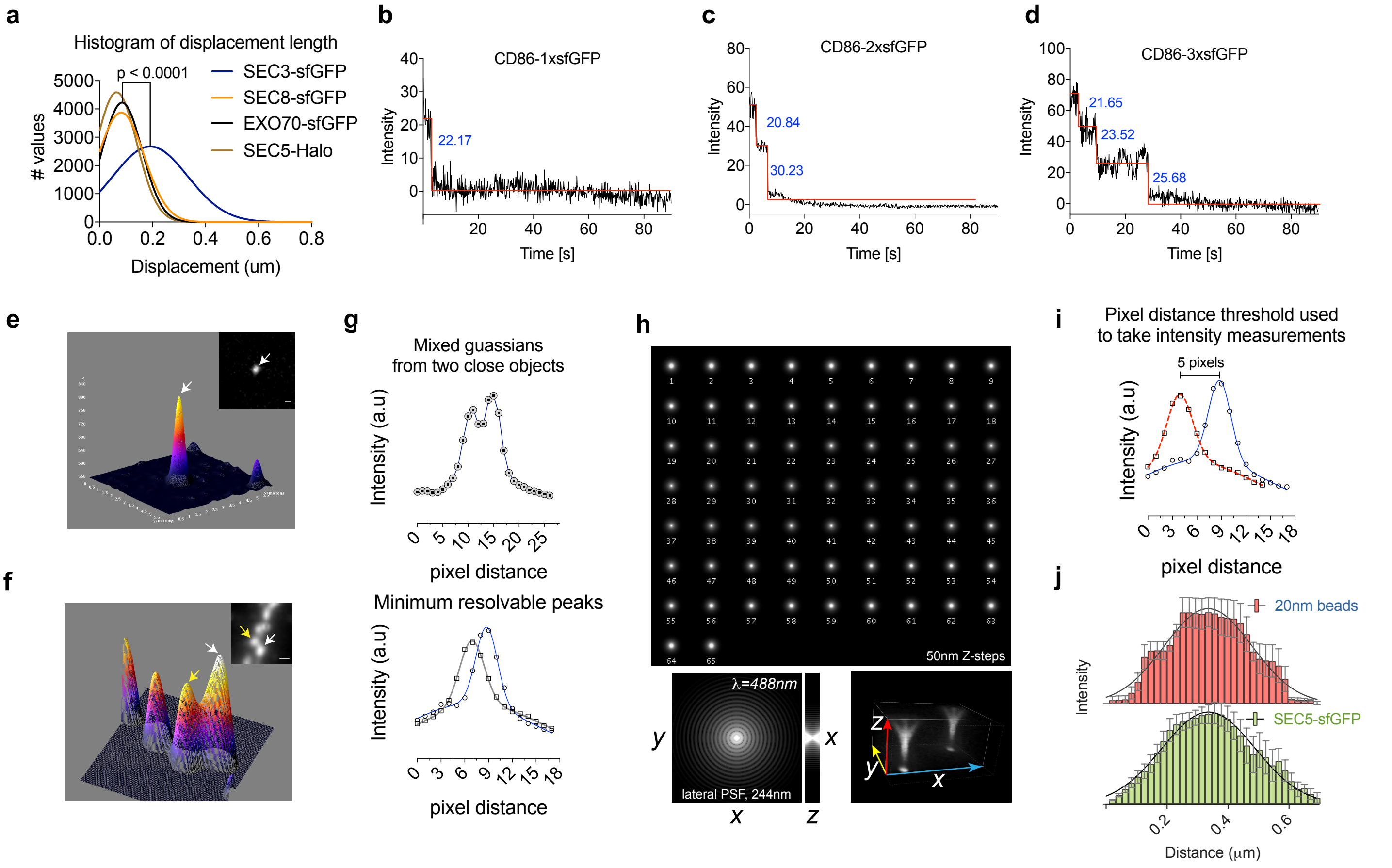

Supplementary Figure 7. Determination of Criteria Used to Count Exocyst Subunit Molecules from Fluorescence Intensities. **(a)** Distribution of vector displacements between the first and last frames of the particle trace. Data are shown from >20,000 object for each exocyst subunit as indicated. Statistical significance was measured by Kruskal-Wallis non-parametric test followed by Dunn's post-hoc test. Photobleaching steps of **(b)** CD86-1xsfGFP **(c)** CD86-2xGFP and **(d)** CD86-3xsfGFP in cells fixed with 3.7% paraformaldehyde followed by methanol and immunolabeled with anti-GFP-biotin antibodies and Streptavidin-ATTO488 dye. **(e)** Fluorescence landscape showing point-spread function of a single tetraspec bead spread on glass. **(f)** Fluorescence landscape of multiple TetraSpeck beads close together. Arrows point to two close but resolvable peaks from which individual intensities could be measured. **(g)** 2D intensity plots of the peaks described in F. **(h)** Empirically determined point spread function of Apo TIRF 60X objective, 1.49NA measured using 20nm TetraSpeck beads. Data shows lateral PSF = 244nm measured at 488nm wavelength, which was used for counting molecules. Pixel size, 120nm per pixel. **(i)** Intensity trace shown in panel is shifted to show minimal separation in pixel distance (5 pixels) that was used to measure intensities of the objects in cells. **(j)** Distributions of average intensity traces over a 20nm bead (n=3) or a SEC5-GFP particle at vesicle fusion sites (n=5).

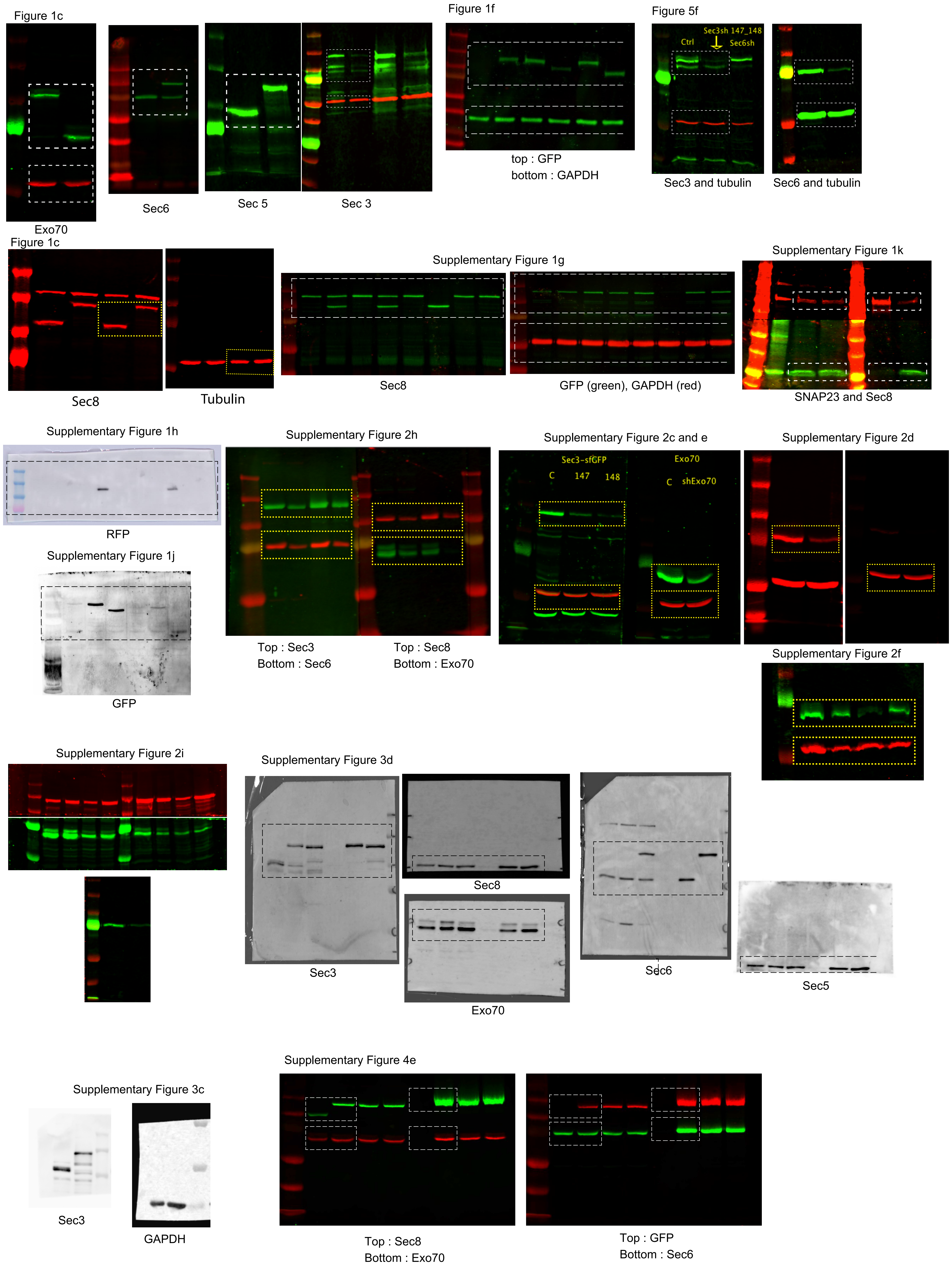

Supplementary Figure 8. Original uncropped images of western blots used in the paper. Each set of immunoblots are annotated with the figure and panel numbers. Where appropriate the region that was cropped out is shown with dashed boxes.

Supplementary Table 1. LC-MS/MS analysis of exocyst subunits interactions

Accession #	Protein Name	Mol wt	Unique peptides			Unique Spectra			Total Spectra			% Coverage		
			EXO70-sfGFP	SEC3-sfGFP	SEC5-sfGFP	EXO70-sfGFP	SEC3-sfGFP	SEC5-sfGFP	EXO70-sfGFP	SEC3-sfGFP	SEC5-sfGFP	EXO70-sfGFP	SEC3-sfGFP	SEC5-sfGFP
A6H5Z3	Exocyst complex component 6B (EXC6B), SEC15L	94 kDa	52	47	48	83	68	69	178	163	116	76	67	68
GFP	Green fluorescent protein	27 kDa	6	7	7	9	12	13	46	35	23	32	34	34
O35250	Exocyst complex component 7 (EXOC7), EXO70	80 kDa	**47**	43	45	**91**	68	71	**239**	167	128	**68**	65	67
O35382	Exocyst complex component 4 (EXOC4), SEC8	111 kDa	53	67	63	93	126	128	237	452	391	72	81	77
Q9D4H1	Exocyst complex component 2 (EXOC2), SEC5	104 kDa	45	57	**54**	65	97	**99**	162	396	**377**	61	68	**69**
Q6KAR6	Exocyst complex component 3 (EXOC3), SEC6	86 kDa	38	52	50	64	88	95	145	472	274	61	68	69
Q8R3S6	Exocyst complex component 1 (EXOC1), SEC3	102 kDa	40	**51**	49	72	**96**	105	145	**372**	268	59	**72**	68
Q3TPX4	Exocyst complex component 5 (EXOC5), SEC10	82 kDa	39	36	35	71	56	55	192	144	94	62	65	63
Q8R313	Exocyst complex component 6 (EXOC6), SEC15	93 kDa	36	30	32	62	42	41	146	95	73	62	53	56
Q6PGF7	Exocyst complex component 8 (EXOC8), EXO84	81 kDa	31	24	27	44	27	27	77	55	49	55	41	45
P63321	RALA	24 kDa	4	4	4	5	4	6	6	5	7	50	40	50
Q9JIW9	RALB	23 kDa	9	6	9	12	6	13	14	7	19	47	33	48
Q8R361	Rab11 family-interacting protein 5 (RFIP5)	70 kDa	5	0	3	5	0	3	5	0	3	10	0	6.2
Q3UPH7	Rho guanine nucleotide exchange factor 40 (ARH40)	165 kDa	7	14	7	7	14	7	7	16	7	5.5	12	5.6
Q91YM2	Rho GTPase-activating protein 35	170 kDa	0	6	0	0	6	0	0	6	0	0	6.3	0
Q60875	Rho guanine nucleotide exchange factor 2(ARHG2)	112 kDa	1	2	0	1	2	0	1	2	0	2	2.2	0
Q9Z0U1	Tight junction protein ZO-2	131 kDa	2	18	13	2	18	13	2	25	14	2.1	17	13
Q811D0	Disks large homolog 1 (DLG1)	100 kDa	12	26	25	12	30	27	14	48	36	20	44	42
O09044	Synaptosomal-associated protein 23 (SNAP23)	23 kDa	6	-	-	6	-	-	6	-	-	39	-	-
Q8BVD5	MAGUK p55 subfamily member 7 (MPP7)	66 kDa	1	6	8	1	6	8	1	7	9	2.1	15	18

Mass spectrometry analysis of tryptic peptides fragments upon GFP-TRAP pull-down of EXO70-sfGFP, SEC3-sfGFP and SEC5-sfGFP from CRISPR knockin NMuMg cell lines. Only peptides determined with > 99%, and protein determined with >95% probability are shown.

Supplementary Table 2. Peptides and primers sequences

1. *Peptides used in MRM-MS*

Uniprot ID	Protein Name	Peptide Sequences
Q8R3S6	SEC3_MOUSE	DLAVVDAK
		LNHFFEGVEAR
Q9D4H1	SEC5_MOUSE	LVLSQLPNFWK
		LFENYIELK
Q6KAR6	SEC6_MOUSE	NIFSVPEIVR
		LTDPSLLYLEVSTLVSK
O35382	SEC8_MOUSE	TYQSITER
		FIQEIEHALGLGPAK
Q3TPX4	SEC10_MOUSE	YFNEFLDGELK
		LSDPSDLPR
Q8R313	SEC15_MOUSE	FPFQDPDLEK
		HAAEGEIYTK
O35250	EXO70_MOUSE	HDFSTVLTVFPILR
		VTDYIAEK
Q6PGF7	EXO84_MOUSE	DFEGAVDLLDK
		QLTEVLVFELSPDR

2. *Primers for genotyping*

Primer #	Exocyst Subunit	Primer direction	Sequence (5' -> 3')
M12	SEC6	Forward	CTGAACGTGGCAAAGCTCCTTA
M13	SEC6	Reverse	GCAGACACAATCCACTCACTGG
M14	SEC6	Forward	GCCTCTGGACATGGCTGTAT
M15	SEC6	Reverse	GTTCATGCTGCCTTCTCTCC
M73	SEC5	Forward	AGCCCGTCACTGTCAGGATG
M74	SEC5	Reverse	ATGGGCATCTTTCTGCACCACC
M75	SEC5	Forward	AGAATTCCATAGGCACTGGCTT
M76	SEC5	Reverse	ACAGGGTACATATGCTGTGCTC
M81	SEC3	Forward	GGTGGTGTGGCACTCCATGC
M82	SEC3	Reverse	CAGCCGCTCGAAAACACAAG
M83	SEC3	Forward	CTGAGGGCCGAGACATGGAG
M84	SEC3	Reverse	CAATGAGCTAATGGGCAGCC
E21	EXO70	Forward	GGTATGGCAGCGTGCCCTTC
E22	EXO70	Reverse	GGGGCTCCTGGTTTGGGCAC

3. *sgRNA sequences to generate knock-ins*

Subunits	sgRNA sequences (5' -> 3') PAM
SEC3	ACTCTTTCCGTAGTGTTTGCTGG
SEC5	AAGAGCATCAGACATGGCGGTGG
SEC6	CCTATGTGTCTTTACTGTGTGG
SEC8	GAGTCTGTGGCCGTGCATACTGG
SEC10	TCTCAGAAGGCCTCGGTAATGGG
SEC15	TATTGTCTCACGACCAGTGCTGG
EXO70	GTGTTTCTGCCCAGTTCTGCCGG
